# CERS6-derived ceramides aggravate kidney fibrosis by inhibiting PINK1-mediated mitophagy in diabetic kidney disease

**DOI:** 10.1152/ajpcell.00144.2023

**Published:** 2023-07-17

**Authors:** Xiangyu Wang, Minkai Song, Xiaomin Li, Cailin Su, Yanlin Yang, Kai Wang, Cuiting Liu, Zongji Zheng, Yijie Jia, Shijing Ren, Wenhui Dong, Jiaqi Chen, Ting Wang, Lerong Liu, Meiping Guan, Chao Zhang, Yaoming Xue

**Affiliations:** ^1^Department of Endocrinology & Metabolism, Nanfang Hospital, Southern Medical University, Guangzhou, People’s Republic of China; ^2^Division of Orthopaedic Surgery, Department of Orthopaedics, Nanfang Hospital, Southern Medical University, Guangzhou, People’s Republic of China; ^3^Division of Hepatobiliopancreatic Surgery, Department of General Surgery, Nanfang Hospital, Southern Medical University, Guangzhou, People’s Republic of China; ^4^Central Laboratory, Southern Medical University, Guangzhou, People’s Republic of China; ^5^Department of Biochemistry and Molecular Biology, School of Basic Medical Science, Guangdong Provincial Key Laboratory of Single Cell Technology and Application, Southern Medical University, Guangzhou, People’s Republic of China

**Keywords:** ceramide synthase 6, diabetic kidney disease, interstitial fibrosis, mitophagy, renal tubular epithelial cell

## Abstract

During diabetic kidney disease (DKD), ectopic ceramide (CER) accumulation in renal tubular epithelial cells (RTECs) is associated with interstitial fibrosis and albuminuria. As RTECs are primarily responsible for renal energy metabolism, their function is intimately linked to mitochondrial quality control. The role of CER synthesis in the progression of diabetic renal fibrosis has not been thoroughly investigated. In this study, we observed a significant upregulation of *ceramide synthase 6* (*Cers6*) expression in the renal cortex of db/db mice, coinciding with increased production of CER (d18:1/14:0) and CER (d18:1/16:0) by Cer6. Concurrently, the number of damaged mitochondria in RTECs rose. *Cers6* deficiency reduced the abnormal accumulation of CER (d18:1/14:0) and CER (d18:1/16:0) in the kidney cortex, restoring the PTEN-induced kinase 1 (PINK1)-mediated mitophagy in RTECs, and resulting in a decrease in damaged mitochondria and attenuation of interstitial fibrosis in DKD. Automated docking analysis suggested that both CER (d18:1/14:0) and CER (d18:1/16:0) could bind to the PINK1 protein. Furthermore, inhibiting *PINK1* expression in *CERS6* knockdown HK-2 cells diminished the therapeutic effect of *CERS6* deficiency on DKD. In summary, CERS6-derived CER (d18:1/14:0) and CER (d18:1/16:0) inhibit PINK1-regulated mitophagy by possibly binding to the PINK1 protein, thereby exacerbating the progression of renal interstitial fibrosis in DKD.

**NEW & NOTEWORTHY** This article addresses the roles of *ceramide synthase 6* (*CERS6*) and CERS6-derived ceramides in renal tubular epithelial cells of diabetic kidney disease (DKD) associated interstitial fibrosis. Results from knockdown of *CERS6* adjusted the ceramide pool in kidney cortex and markedly protected from diabetic-induced kidney fibrosis in vivo and in vitro. Mechanically, CERS6-derived ceramides might interact with PINK1 to inhibit PINK1/Parkin-mediated mitophagy and aggravate renal interstitial fibrosis in DKD.

## Graphical abstract

**Figure:**
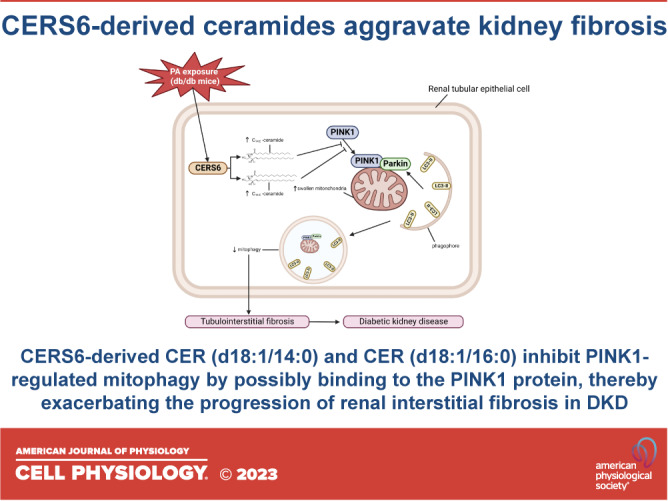

## INTRODUCTION

In patients with diabetes, diabetic kidney disease (DKD) frequently presents as a common complication. Global epidemiological surveys of diabetes-related end-stage renal disease (ESRD) have demonstrated that DKD has emerged as the predominant cause of ESRD (1). Investigating the underlying mechanisms to delay the onset and progression of DKD holds considerable significance in alleviating the burden on public healthcare systems.

In previous studies, ectopic ceramide (CER) accumulation has been observed in the kidneys of patients with DKD and experimental animal models, primarily in renal tubular epithelial cells (RTECs) (2, 3). Intracellular CER accumulation is associated with altered RTEC morphology, such as flattening, brush border loss, and interstitial fibrosis (4, 5). CER and its metabolites have been shown to negatively impact insulin sensitivity, islet β-cell activity, vascular responsiveness, and mitochondrial function (6). Inhibition of CER synthesis or promotion of CER degradation ameliorates various metabolic abnormalities associated with diabetes mellitus, cardiomyopathy, insulin resistance, atherosclerosis, and fatty liver disease (7, 8). Given that DKD shares numerous risk factors with the aforementioned diseases, emerging studies have demonstrated CER as an important contributing factor (9, 10).

It remains an unmet need to investigate the pathogenic mechanism of CER accumulation in DKD. Although CER accumulation is known to be detrimental to the progression of DKD, complete inhibition of ceramide synthesis may still result in potential side effects (11, 12). The suppression of the rate-limiting enzyme in the de novo synthesis of CER may result in embryonic lethality in mice (13). Six ceramide synthases (CerS1-6) generate CER with specificity in acyl-chain lengths. These enzymes exhibit substrate specificity and diverse tissue distribution, forming distinct CER pools (6). This allows for the investigation of the regulation of specific CER-dependent signaling pathways. Notably, plasma levels of CER (d18:1/16:0) are significantly elevated in patients with DKD (14); however, the role of CER (d18:1/16:0) in renal fibrosis remains unclear. Since CER (d18:1/16:0) is produced by CERS6 (6), it is essential to determine whether CERS6 regulates DKD development by producing CER (d18:1/16:0), verify its underlying mechanism, and identify its potential as a therapeutic target.

This study addresses the roles of CERS6 and CERS6-derived CER during DKD-associated interstitial fibrosis. This mechanism lays the groundwork for future therapeutic strategies for patients with DKD, particularly those with abnormal lipid metabolism.

## MATERIALS AND METHODS

### Animal Model

All study protocols for animal experiments were approved by the Nanfang Hospital Animal Ethic Committee (NFYY-2022-0367) and followed the National Research Council’s Guide for the Care and Use of Laboratory Animals. GemPharmatech (T002407, Nanjing, China) provided diabetic db/db mice on a C57BLKS/J background (RRID:IMSR_JAX:000662, 7 wk of age, male) as well as non-diabetic db/m mice (7 wk of age, male). The mice were kept in a specific pathogen-free room and were given a normal diet. The mice were weighed, and their blood glucose levels were measured every 2 wk. Mouse albumin ELISA quantitation kit (MM-0705M2, Meimian, Jiangsu, China) was used to determine the total amount of albumin in 24 h of urine collected from mice in the metabolic cage at the age of 20 wk. After the mice were euthanized, part of the kidney specimens was transferred to 4% paraformaldehyde (PFA) (BL539A, Biosharp, Beijing, China) for paraffin embedding and sectioning, and part of the cortex was placed in 2.5% glutaraldehyde (DF0151, LEAGENE, Beijing, China) for subsequent transmission electron microscopy (TEM) experiments, while the rest were rapidly frozen in liquid nitrogen for subsequent lipid extraction and molecular biological detection.

### Generation of Lentivirus Vectors

Lentivirus was used for the construction of the *CERS6* knockdown model in vitro and in vivo. We transfected pSPAX2 and pMD2.G with plasmids containing the sequences for knockdown of *CERS6* or scrambled control into 293 T cells (20181122-01, Suyan, Guangzhou, China) using Lipo8000 (C0533, Beyotime, Beijing, China). After collection and filtration, the supernatants were used directly for cell infecting or concentrated by lentivirus concentration reagent (BF06205, Biodragon, Suzhou, China) for mouse experiments. Tsingke Biotechnology (Beijing, China) designed and produced the plasmids, and the shRNA sequence is presented in Table 1.

**Table 1.** shRNA and siRNA sequences

Name	Sequence (5′–3′)
*Homo-CERS6-shRNA*	CCGGGCTCATCTTCGAGAGATTTGTCTCGAGACAAATCTCTCGAAGATGAGCTTTTTGAATT
*Mus-Cers6-shRNA*	CCGGGCTATAACAAAGCATCCTGATCTCGAGATCAGGATGCTTTGTTATAGCTTTTTT
*Homo-PINK1-siRNA*	GAGACCTGAAATCCGACAA

### Generation of *Cers6* Knockdown Mice

Lentivirus was prepared at a dose of 6 × 10^7^ TU per mouse, according to the method described by Yang et al. (15). Six 8-wk-old db/db mice were randomly divided into two groups to receive either the *Cers6*-knockdown lentivirus (db/db + *Cers6* shRNA) or empty control plasmid (db/db + Vector shRNA) via tail vein injection. The same amount of empty control plasmid (db/m + Vector shRNA) was administered to three age-matched db/m mice. To have a sustained genetic deficiency effect, the injection was given at ages 8, 12, and 16 wk.

### Histology and Immunostaining

Paraffin-embedded kidney tissues were shaved into 4-μm thick sections for morphological examination. Sections were dewaxed to water and stained with hematoxylin and eosin (H&E), Periodic acid-Schiff (PAS), and MASSON according to kit instructions (G1005, G1008, G1006, Servicebio, Wuhan, China). Performed a heat-mediated antigen retrieval with ethylene diamine tetraacetic acid (PH9.0) (P0085, Beyotime, Shanghai, China) before commencing with immunostaining protocol. After blocking endogenous peroxidase with 3% hydrogen peroxide solution (G0115, Servicebio, Wuhan, China), the tissue was blocked with 3% bull serum albumin (BSA) (G5001, Servicebio, Wuhan, China) to prevent non-specific binding of the antibody. A diluted primary antibody was applied to the surface of the tissue, and the sections were incubated at 4°C overnight. The secondary antibodies were dripped onto the tissues and incubated at room temperature for an hour after the sections had been brought back to room temperature. During immunohistochemistry (IHC), diaminobenzidine (G1211, Servicebio, Wuhan, China) was used for color development. Therefore, nuclei in tissues were counterstained with hematoxylin (G1004, Servicebio, Wuhan, China) in the IHC or with DAPI (G1012, Servicebio, Wuhan, China) in the immunofluorescence (IF). The sections stained by H&E, PAS, MASSON, or IHC were dehydrated and sealed with neutral gum (WG10004160, Servicebio, Wuhan, China), and the results were collected and interpreted under a light microscope (Nikon, Japan) at ×400. Sections for IF were conducted with an antifade mounting medium (P0126, Beyotime, Shanghai, China) and a LSM880 laser scanning confocal microscope (ZEISS, Germany) at a magnification of ×200. Information about antibodies related to this experiment is listed in Table 2.

**Table 2.** Antibodies used in this study

Antibodies	Company	Cat. No.	Concentration	Validated Information
Anti-Collagen I	abcam	ab279711	IHC 1:100WB 1:1,000	https://www.abcam.cn/products/primary-antibodies/collagen-i-antibody-epr24331-53-bsa-and-azide-free-ab279711.html
Anti-CERS6	Santa Cruz	sc-100554	1:1,000	https://www.scbt.com/p/lass6-antibody-l-18?requestFrom=search
Anti-α-SMA	Bioss	bs-10196R	1:1,000	http://bioss.com.cn/prolook_03.asp?id=AF08169606017271&pro37=1
Anti-P62	Proteintech	18420-1-AP	1:1,000	https://www.ptgcn.com/products/SQSTM1-Antibody-18420-1-AP.htm
Anti-Parkin	Proteintech	14060-1-AP	1:1,000	https://www.ptgcn.com/products/PARK2-Antibody-14060-1-AP.htm
Anti-PINK1	Proteintech	23274-1-AP	1:1,000	https://www.ptgcn.com/products/PINK1-Antibody-23274-1-AP.htm
Anti-LC3	Proteintech	14600-1-AP	1:1,000	https://www.ptgcn.com/products/MAP1LC3B-Antibody-14600-1-AP.htm
AF488-conjugated anti-LC3B	Bioss	bs-4843R-AF488	1:100	http://bioss.com.cn/prolook_03_biaoji.asp?pro2a=20121219200384843&pro33=216
Anti-TOM20	Beyotime	AF1717	1:100	https://www.beyotime.com/product/AF1717.htm
Anti-β-tubulin	Abmart	M20045	1:2,000	http://www.ab-mart.com.cn/page.aspx?node=%2059%20&id=%2049688
Anti-COXIV	abcam	ab202554	1:2,000	https://www.abcam.cn/products/primary-antibodies/cox-iv-antibody-epr9442abc-mitochondrial-loading-control-ab202554.html
HRP-conjugated Goat anti-rabbit IgG	Proteintech	SA00001-2	IHC 1:200WB 1:5,000	https://www.ptgcn.com/products/HRP-conjugated-Affinipure-Goat-Anti-Rabbit-IgG-H-L-secondary-antibody.htm
HRP-conjugated Goat anti-mouse IgG	Proteintech	SA00001-1	1:5,000	https://www.ptgcn.com/products/HRP-conjugated-Affinipure-Goat-Anti-Mouse-IgG-H-L-secondary-antibody.htm
Cy3-conjugated Goat anti-rabbit IgG	Servicebio	GB21303	1:200	https://www.servicebio.cn/goodsdetail?id=253

### Transmission Electron Microscopy

Transmission electron microscopy (TEM) was used to detect mitochondrial morphology and autophagosomes in the renal cortex of mice following gene therapy, according to the method of Han et al. (16) and Li et al. (17). Fresh renal cortices were rapidly cut into 1 mm^3^ sections and immediately placed in a pre-cooled 2.5% glutaraldehyde solution (DF0151, LEAGENE, Beijing, China) at 4°C. Tissues were embedded and cut into 60–80 nm thick sections for transmission electron microscope (Hitachi, Japan) at ×3,000 and ×8,000.

### Lipids Extraction and Quantification of CER from the Kidney Cortex

Lipid extraction and quantification of CER from the kidney cortex were performed as precedently described with modifications (18). Shortly, tissues were added to sterile saline and completely smashed to homogenate by an automatic sample grinding instrument (Jingxin, Shanghai, China). Lipids from tissue homogenates were extracted with mass spectral level isopropanol (I811932, MACKLIN, Shanghai, China). CER levels were determined by HPLC-ESI-MS/MS performed on TSQ Quantiva LC-MS/MS system (Thermo Fisher Scientific). The protein concentration of the homogenates routinely proceeded for relative quantification.

### Culture and Treatment of Cells

An epithelial cell line derived from the proximal tubule of the human kidney, HK-2 cells (HTX2165, Otwo Biotech, Shenzhen, China), was grown in DMEM/F12 (11320082, Gibco, Carlsbad, CA) supplemented with 10% fetal bovine serum (10100147, Gibco, Carlsbad, CA) at 37°C and 5% CO_2_. Polybrene (C0351, Beyotime, Beijing, China) at 5 g/mL was used to infect HK-2 cells with control lentivirus vectors (LV-sh-NC) or *CERS6* shRNA lentivirus vectors (LV-sh-*CERS6*). Then, infected HK-2 cells were re-seeded in DMEM/F12 and added with puromycin (ST551, Beyotime, Beijing, China) for 7 days. In addition, lentivirus-infected HK-2 cells were transfected with PTEN-induced kinase 1 (PINK1) siRNA (si-*PINK1*) or control plasmids (si-NC) by Lipo8000 (C0533, Beyotime, Beijing, China) for gene disruption. An analysis of the efficiency of infection and transfection was conducted using reverse transcription-quantitative polymerase chain reaction (RT-qPCR). In accordance with Feng’s protocol (19), the palmitic acid (PA) (P0500, Sigma-Aldrich, Darmstadt, Germany) in 0.01 M NaOH (GC107001, Servicebio, Wuhan, China) was heated at 70°C for 30 min to dissolve and then mixed with 5% BSA (G5001, Servicebio, Wuhan, China) in phosphate buffer saline (ST476, Beyotime, Beijing, China) at a ratio of 3:1 M. Before the 48-h stimulation of control BSA (BSA-CON) or BSA-PA (300 μM), cells were deprived of serum for 24 h. The sequence of siRNA is listed in Table 1. All cell lines used in this study were identified by the manufacturers using short tandem repeat profiling.

### Cellular IF

The evaluation of mitophagy by the cellular IF method was based on Li et al. (20). Lentivirus-infected HK-2 cells were seeded in the middle of the confocal dishes, stimulated with BSA-PA or BSA-CON, then incubated with100nM Mito-Tracker Red CMXRos (C1035, Beyotime) solution. A 4% PFA solution (BL539A, Biosharp, Beijing, China) was used to fix the cells, methanol (M813895, MACKLIN, Shanghai, China) was used to permeabilize them, and 3% BSA (G5001, Servicebio, Wuhan, China) was used to block them. The cells were then incubated at 4°C overnight with LC3B/AF488 conjugated antibody (1:100, bs-4843R-AF488, Bioss, Beijing, China). After staining with DAPI (G1012, Servicebio, Wuhan, China), cells were analyzed using an FV3000 confocal laser scanning microscope (Olympus, Japan) at a magnification of ×1,000.

### RNA Extraction and RT-qPCR

The total RNA was isolated from kidney cortex or HK-2 cells using TRIzol reagent (15596026CN, Life Technologies, CA), according to the manufacturer’s instructions. Following the verification of RNA concentration and purity by Nanodrop 2000 (Thermo Fisher Scientific), reverse transcription of mRNA was performed using the PrimeScript reverse transcription kit (RR014A, Takara, Shiga, Japan). The RT-qPCR reactions were performed using Hieff qPCR SYBR Green Master Mix (11202ES08, Yeasen Biotechnology, Shanghai, China) and QuantStudio 3 (Applied Biosystems, CA). Primer sequences for mRNA expression are listed in Table 3.

**Table 3.** Primer sequences

Name	Forward	Reverse
*mCers1*	CCACCACACACATCTTTCGG	GGAGCAGGTAAGCGCAGTAG
*mCers2*	ATGCTCCAGACCTTGTATGACT	CTGAGGCTTTGGCATAGACAC
*mCers4*	TACCCACATCAGACCCTGAAT	TGAAGTCCTTGCGTTTGACATC
*mCers5*	TGCTGTTTGAGCGATTTATTGC	GGTTCCACCTTATTGACAGGAC
*mCers6*	GATTCATAGCCAAACCATGTGCC	AATGCTCCGAACATCCCAGTC
*mActb*	GGCTGTATTCCCCTCCATCG	CCAGTTGGTAACAATGCCATGT
*hCERS1*	TGTGGGCATCCTTGTGCTC	GAGGCGGAACCAGAACCAG
*hCERS2*	GCTCTTCCTCATCGTTCGATAC	CTTGCCACTGGTCAGGTAGA
*hCERS4*	CTGGTGGTACCTCTTGGAGC	CGTCGCACACTTGCTGATAC
*hCERS5*	TCGCCATCGGAGGAATCAG	CCAAGGTGACGACCAGAGAAA
*hCERS6*	GGACCACAAATTGCTCCGC	GGCTTCTCCTGATTGCGTCT
*hACTB*	CATGTACGTTGCTATCCAGGC	CTCCTTAATGTCACGCACGAT

### Protein Extraction and Western Blotting

Before the protein analysis process, mitochondria were extracted from renal cortex and HK-2 cells using mitochondrial extraction kits (C3606, C3601, Beyotime, Beijing, China), and then RIPA Lysis Buffer (P0013K, Beyotime, Beijing, China) with 1% protease (P0015, Beyotime, Beijing, China) and 1% phosphatase inhibitor (P1081, Beyotime, Beijing, China) were used to extract proteins in mitochondria or whole cells. BCA kit (P0012, Beyotime, Beijing, China) was used to measure protein concentration, and lysis and loading buffers (P0015, Beyotime, Beijing, China) were used to dilute the concentration. Then the proteins were denatured by heating in a 98°C water bath. We separated the same amounts of proteins using precast SDS-PAGE gels (4%–15% or 15%; P0466, P0462, Beyotime, Beijing, China). Following the isolation of the proteins, they were transferred onto polyvinylidene fluoride membranes (IPVH00010, Millipore, MA), as previously described (21). The membranes were blocked with QuickBlock Blocking Buffer for Western blot (P0252, Beyotime, Beijing, China) and incubated overnight at 4°C with primary antibodies. As soon as the membranes had been washed, they were incubated with secondary antibodies conjugated with HRP (Proteintech, IL) and detected with a BeyoECL kit (P0018S, Beyotime, Beijing, China) performed by Tanon 2500 automatic chemiluminescence image analysis system (Tanon, Shanghai, China). ImageJ was used to quantify the intensity of the bands. Information about antibodies related to this experiment is listed in Table 2.

### Computational Docking

According to the protocol of computational docking provided by AutoDock suite (22), CER (d18:1/14:0), and CER (d18:1/16:0) were docked to PINK1 protein, respectively, using Autodock Vina 1.1.2 (Center for Computational Structural Biology, CA). PyMol 2.3.0 (Schrödinger, LLC) was applied to visualize the interaction mode of the docking results.

### Statistical Analysis

GraphPad Prism8.0 (GraphPad Software, CA) was an app for data analysis and image production. For continuous variables with a normal distribution, means ± SD was used, and differences between groups were compared by means of a Student’s *t* test or a Welch’s *t* test. For data with a non-Gaussian distribution, a Mann–Whitney *U* test was used to compare the two groups. Several comparisons between selected groups were conducted using a one-way ANOVA. There were at least three replicates for each experiment, and *P* < 0.05 was considered significant for each experiment.

## RESULTS

### Upregulated *Cers6* and Cers6-Derived CER Were Found in the Kidney Cortex of Diabetic Mouse Models

**Figure 1.**
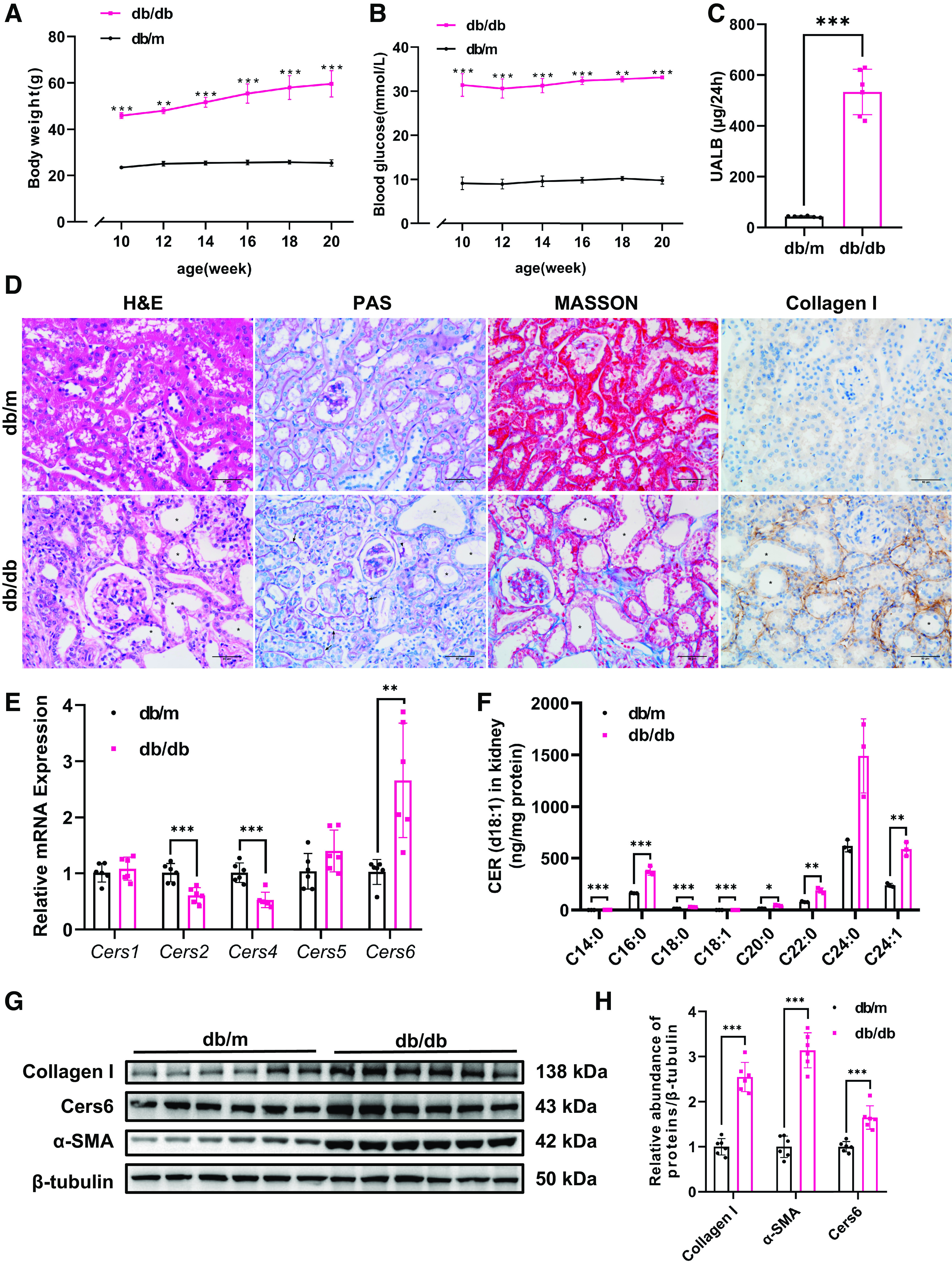
Abnormal CER metabolism in the kidney of diabetic mouse models. Body weight (*A*) and blood glucose (*B*) in db/db mice (pink) and db/m mice (black) were measured at the indicated weeks of age. Data are shown as means ± SD, *n* = 6, **P < 0.01, ***P < 0.001 between db/db and db/m at the same time point. *C*: 24 h of albuminuria were measured at 20 wk. Data are shown as means ± SD, *n* = 6, ***P < 0.001. *D*: representative images of H&E, PAS, MASSON, and immunohistochemical staining for Collagen I in kidneys (*n* = 3). Star indicates tubular atrophy. The black arrow indicates basement membrane thickening. The white arrow indicates collagen deposition. Triangle indicates protein droplets in the cytoplasm of renal tubular epithelium. The bar indicates 50 μm. *E*: analysis of ceramide synthesis mRNA expression in the kidney cortices of db/db mice and db/m mice. Data are shown as means ± SD, *n* = 6, **P < 0.01, ***P < 0.001. *F*: CER levels in the kidney cortices of db/db mice and db/m mice. Data are shown as means ± SD, *n* = 3, *P < 0.05, **P < 0.01, ***P < 0.001. *G*, *H*: Western blots and quantification of Collagen I, α-SMA, and Cers6 in the kidney cortices of db/db mice and db/m mice. Data are shown as means ± SD, *n* = 6, ***P < 0.001. CERS6, ceramide synthase 6; CER, ceramide; H&E, hematoxylin and eosin; PAS, Periodic acid-Schiff.

To explore the specific CER species that contribute to interstitial fibrosis in DKD, we first measured the CER species and the expression profiles of ceramide synthesis in the kidney cortex of a diabetic mouse model (db/db mice) versus control. A significant increase in body weight (Fig. 1*A*), blood glucose (Fig. 1*B*), and 24-h albuminuria (Fig. 1*C*) was observed in db/db mice. Tubular atrophy, thickened tubule basement membrane, protein droplets, and collagen deposition can be observed in the kidneys of db/db mice (Fig. 1*D*). We then performed HPLC-ESI-MS/MS for CER species in the kidney cortex of each group. Also, we examined the expression of ceramide synthesis mRNA in the kidney cortex of db/db and control mice. We found that elevated *Cers6* mRNA expression was accompanied by an increase of CER (d18:1/14:0) and CER (d18:1/16:0) in the kidney cortex of diabetic mice (Fig. 1, *E* and *F*). A Western blot analysis was conducted to further verify the above experiments’ findings. In diabetic mice, the expression of Collagen I, alpha smooth muscle Actin(α-SMA), and Cers6 was elevated as expected (Fig. 1, *G* and *H*). The results above indicate that Cers6 and Cers6-derived CER were enhanced in diabetic kidney disease.

### Knockdown of *Cers6* Regulated CER Metabolism and Reduced Tubulointerstitial Fibrosis in db/db Mice

**Figure 2.**
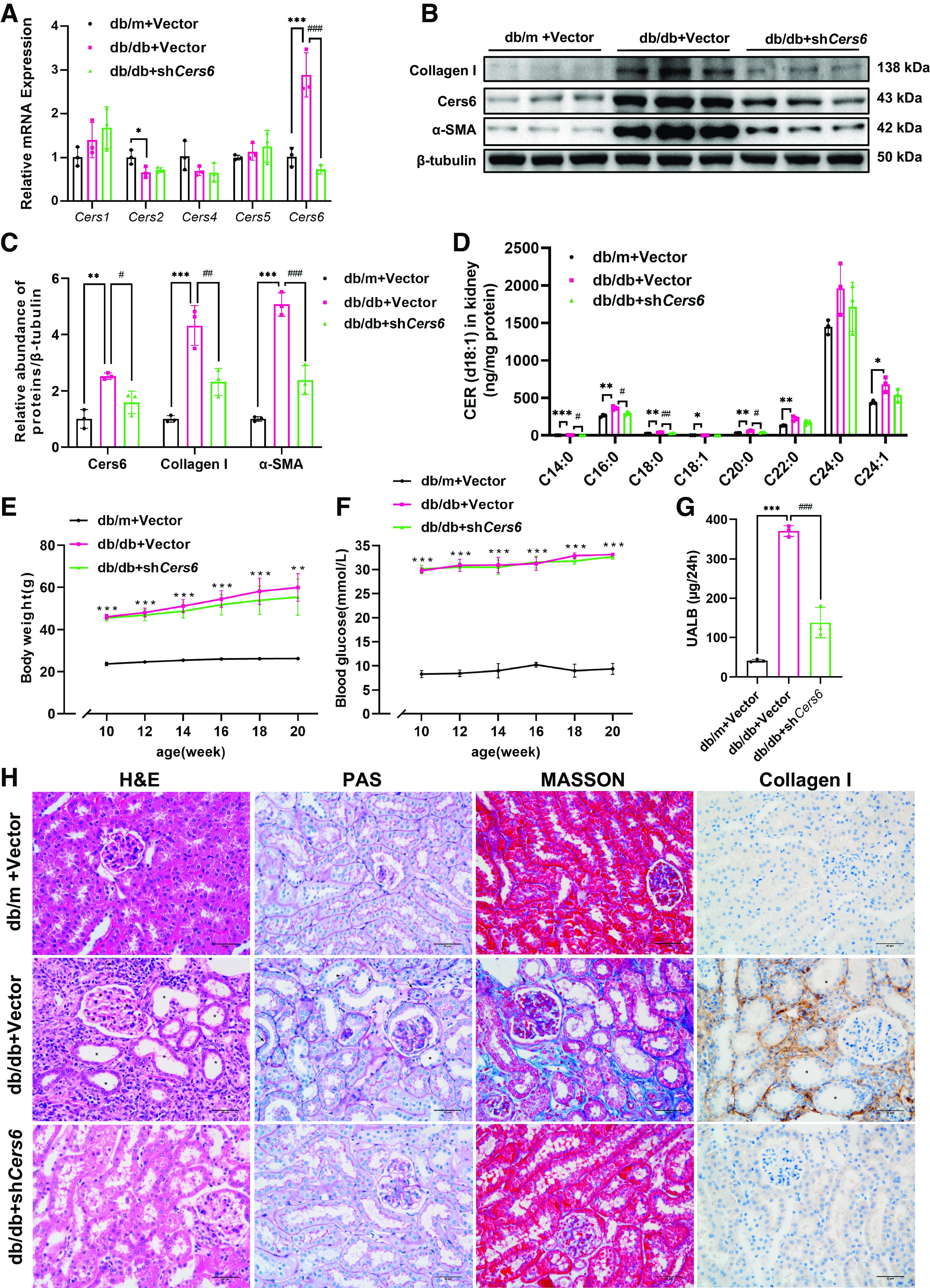
Knockdown of *Cers6* in db/db mice regulated CER metabolism and reduced tubulointerstitial fibrosis. *A*: analysis of ceramide synthesis mRNA expression in the kidney cortices of db/db + *Cers6* shRNA, db/db + vector shRNA, and db/m + vector shRNA. Data are shown as means ± SD, *n* = 3, *P < 0.05, ***P<0.001 vs. db/m + vector shRNA, ###*P* < 0.001 vs. db/db + *Cers6* shRNA. *B*, *C*: Western blots and quantification of Collagen I, α-SMA, and Cers6 in the kidney cortices of each group. Data are shown as means ± SD, *n* = 3, **P < 0.01, ***P < 0.001 vs. db/m + vector shRNA, #*P* < 0.05, ##*P* < 0.01, ###*P* < 0.001 vs. db/db + *Cers6* shRNA. *D*: CER levels in the kidney cortices of each group. Data are shown as means ± SD, *n* = 3, ***P < 0.001 vs. db/m + vector shRNA, ###*P* < 0.001 vs. db/db + *Cers6* shRNA. Body weight (*E*), and blood glucose (*F*) in db/db + *Cers6* shRNA (green triangle), db/db + vector shRNA (pink square), and db/m + vector shRNA (black circle) were measured at the indicated weeks of age. Data are shown as means ± SD, *n* = 3, **P < 0.01, ***P < 0.001 between db/db + vector shRNA and db/m + vector shRNA at the same time point. *G*: Twenty-four hours of albuminuria were measured at 20 wk. Data are shown as means ± SD, *n* = 3, ***P < 0.001 vs. db/m + vector shRNA, ###*P* < 0.001 vs. db/db + *Cers6* shRNA. *H*: representative images of H&E, PAS, MASSON, and immunohistochemical staining for Collagen I in kidneys. Star indicates tubular atrophy. The black arrow indicates basement membrane thickening. The white arrow indicates collagen deposition. Triangle indicates protein droplets in the cytoplasm of renal tubular epithelium. The bar indicates 50 μm. CERS6, ceramide synthase 6; CER, ceramide; H&E, hematoxylin and eosin; PAS, Periodic acid-Schiff.

To further investigate the role played by CERS6 in diabetic kidney disease, a lentivirus containing a *Cers6* shRNA was injected into the tail vein of db/db mice to silence the expression of *Cers6*. An analysis of *Cers6* mRNA and protein abundance in the renal cortex of db/db mice showed that *Cers6* was reduced in a diabetic mouse model after *Cers6* silencing (Fig. 2, *A*–*C*). Cers6-derived CER (d18:1/14:0) and CER (d18:1/16:0) decreased by *Cers6* knockdown (Fig. 2*D*). Although the knockdown of *Cers6* did not significantly reduce body weight (Fig. 2*E*) or blood glucose (Fig. 2*F*) in mice, mice that received lentiviruses containing a *Cers6* shRNA exhibited reduced albuminuria (Fig. 2*G*). Moreover, histopathologic changes in the kidney (Fig. 2*H*) and Western blot analysis of the renal cortex (Fig. 2, *B* and *C*) demonstrated that blocking *Cers6* could alleviate tubulointerstitial lesions. Collectively, deficiency of *Cers6* adjusted the CER pool in the kidney cortex and markedly protected it from diabetic-induced kidney fibrosis.

### Knockdown of *Cers6* Ameliorated Mitophagy and Reduced Damaged Mitochondria in Renal Tubules of Diabetic Mice

**Figure 3.**
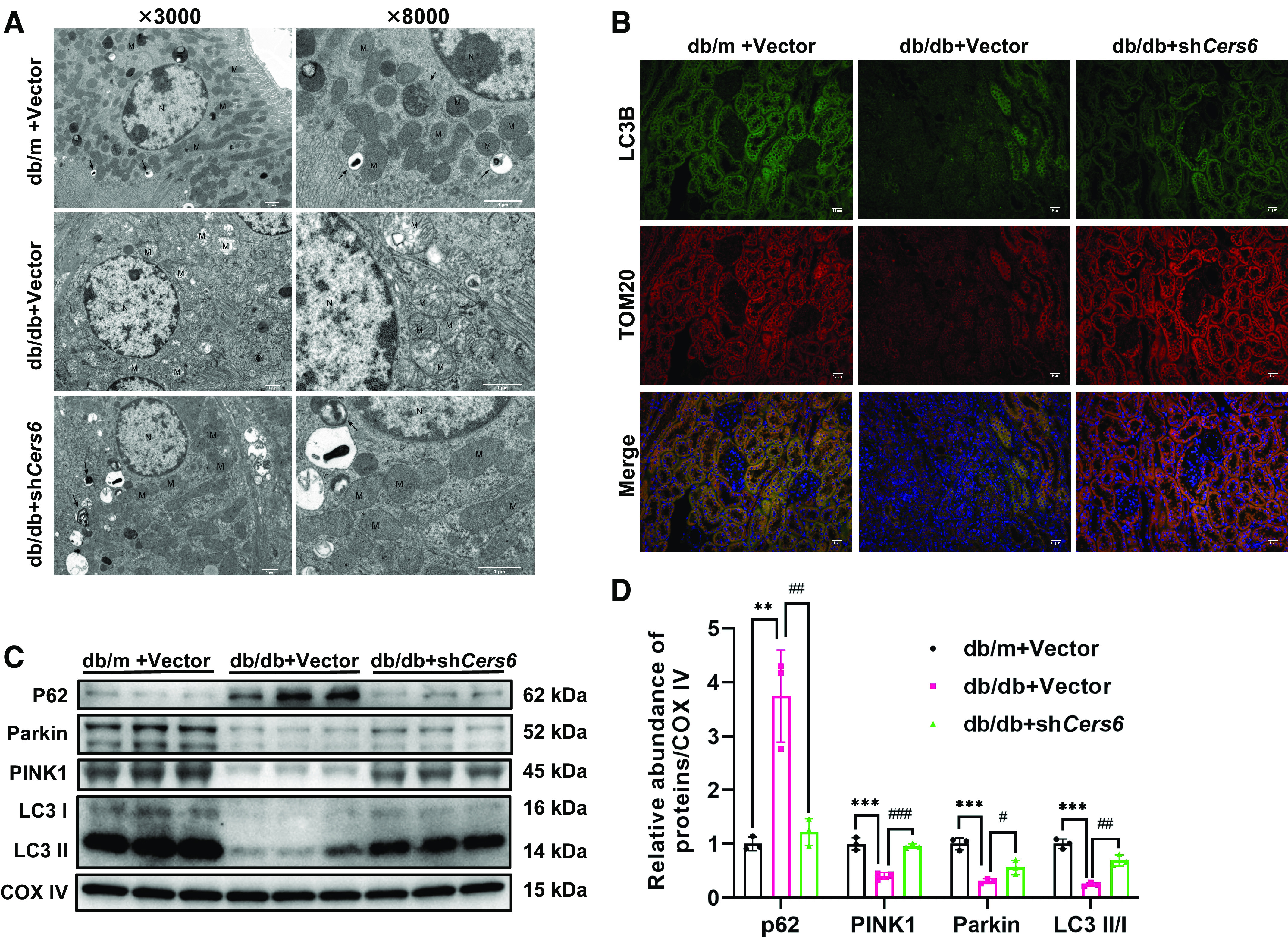
Knockdown of *Cers6* in db/db mice restored mitophagy and reduced damaged mitochondria in renal tubules. *A*: mitochondria and autophagosomes in proximal tubules were examined by TEM (*n* = 3). Arrows indicate the autophagosomes; M, mitochondria; N, nucleus. The bar indicates 1 μm. *B*: representative immunofluorescence images of LC3B (green) and TOM20 (red) in kidneys. The nuclei were counterstained by DAPI (blue) (*n* = 3). The bar indicates 10 μm. *C*, *D*: Western blots and quantification of p62, PINK1, Parkin, and LC3 in the mitochondria isolated from kidney cortices of db/db + *Cers6* shRNA, db/db + vector shRNA and db/m + vector shRNA. Data are shown as means ± SD, *n* = 3, **P < 0.01, ***P < 0.001 vs. db/m + vector shRNA, #*P* < 0.05, ##*P* < 0.01, ###*P* < 0.001 vs. db/db + *Cers6* shRNA. CERS6, ceramide synthase 6; PINK1, PTEN-induced kinase 1; TEM, transmission electron microscopy.

The kidney is the second largest energy-consuming organ of the human body, among which renal tubular epithelial cells are the major consumers of adenosine triphosphate, which is closely related to the generation of mitochondrial energy. As Fig. 3*A* demonstrated, TEM detection revealed more mitochondria deformations of proximal tubular cells and fewer autophagosomes in diabetic mice than in the control. Interestingly, these changes were significantly reversed by *Cers6* knockdown (Fig. 3*A*). Since autophagosomes seem to be involved in the above process, we attempted to evaluate whether mitophagy would be affected after knocking down *Cers6* in db/db mice. Kidney sections were co-immunostained for LC3B and TOM20 to indicate mitophagy. Colocalization analysis between punctate LC3B and TOM20 showed that the knockdown of *Cers6* in db/db mice restored the inhibited mitophagy in the diabetic kidney (Fig. 3*B*). Consistent with morphological changes, mitophagy-related protein expression in each group’s mitochondria was assessed by Western blot. The abundances of PINK1, Parkin, and LC3 II/I were markedly decreased and p62 was significantly elevated in diabetic mice (Fig. 3*C*). While the above protein levels were partially restored after the knockdown of *Cers6* (Fig. 3*D*). Taken together, these experiments suggest that down-regulation of *Cers6* expression in vivo can reverse the inhibited mitophagy and restore mitochondrial morphology in diabetic kidney disease.

### Knockdown of *CERS6* May Restore PINK1-Mediated Mitophagy by Regulating CER Metabolism and Improving Tubulointerstitial Fibrosis in PA-Induced HK-2 Cells

**Figure 4.**
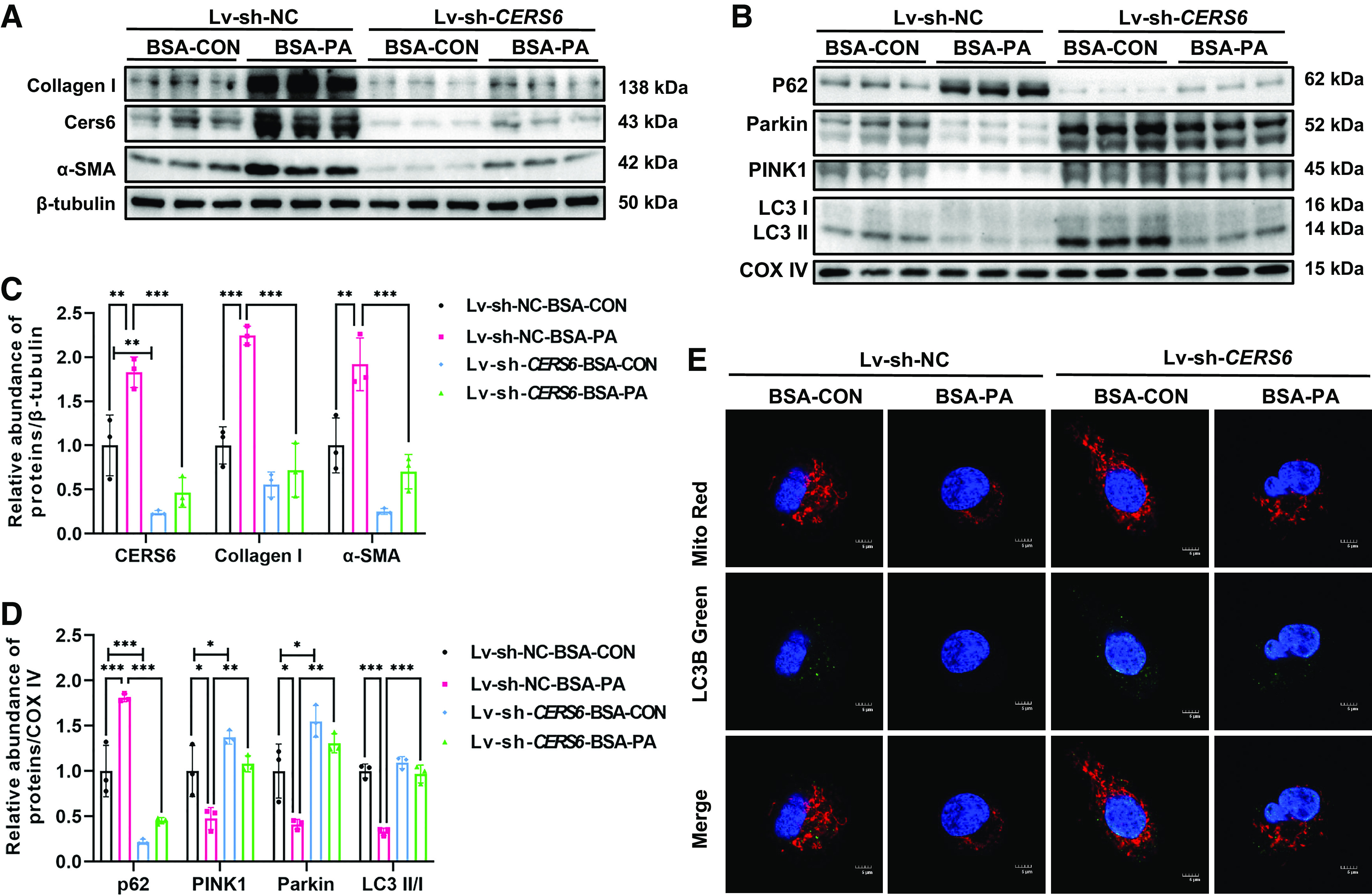
Knockdown of *CERS6* in HK-2 cells ameliorated PA-induced mitophagy dysfunction and renal tubulointerstitial fibrosis. *A*, *C*: Western blots and quantification of Collagen I, α-SMA, and CERS6 in HK-2 cells transfected with empty vector (sh-NC) or *CERS6* shRNA(sh-*CERS6*) plasmid under BSA or BSA-PA conditions. Data are shown as means ± SD, *n* = 3, **P < 0.01, ***P < 0.001. *B*, *D*: Western blots and quantification of p62, PINK1, Parkin, and LC3 in mitochondria isolated from HK-2 cells under each condition. Data are shown as means ± SD, *n* = 3, *P < 0.05, **P < 0.01, ***P < 0.001. *E*: HK-2 cells under each condition were immunostained with MitoTracker (red) and LC3B (green) to show mitophagy (n = 3). The bar indicates 5 μm. BSA, bull serum albumin; CERS6, ceramide synthase 6; PINK1, PTEN-induced kinase 1; PA, paraformaldehyde.

In addition, we used lentivirus to construct stable *CERS6*-deficient HK-2 cells and stimulated them with palmitic acid (PA) to validate whether knockdown of *CERS6* could rescue PA-induced effects in vitro. First, PA exposure strongly induce protein expression of CERS6 matched with an increase of fibrosis-related proteins in HK-2 cells (Fig. 4, *A* and *C*). Second, co-staining with mitochondrial and autophagosome marker LC3B in HK-2 cells demonstrated a reduction of autophagy and mitophagy in response to PA stimulation (Fig. 4*E*). Similar to the in vivo results, *CERS6*-deficient HK-2 cells were significantly less affected by PA stimulation. This was reflected in lower intracellular CERS6 expression matched with fewer accumulation fibrosis-related proteins compared with PA-stimulated non-knockdown HK-2 cells (Fig. 4, *A* and *C*). Consistently, higher levels of PINK1, Parkin, and LC3 II/I protein and lower amount of P62 in mitochondria were accompanied by more mitochondria colocalized with LC3B were detected in PA-stimulated *CERS6*-deficient HK-2 cells compared with control (Fig. 4, *B*, *D*, and *E*). Here, we found that inhibition of *CERS6* may restore PINK1-mediated mitophagy by regulating CER metabolism and relieving fibrosis in PA-induced HK-2 cells.

### CERS6-Derived CER May Bind to PINK1 and Inhibit Mitophagy Mediated by PINK1

**Figure 5.**
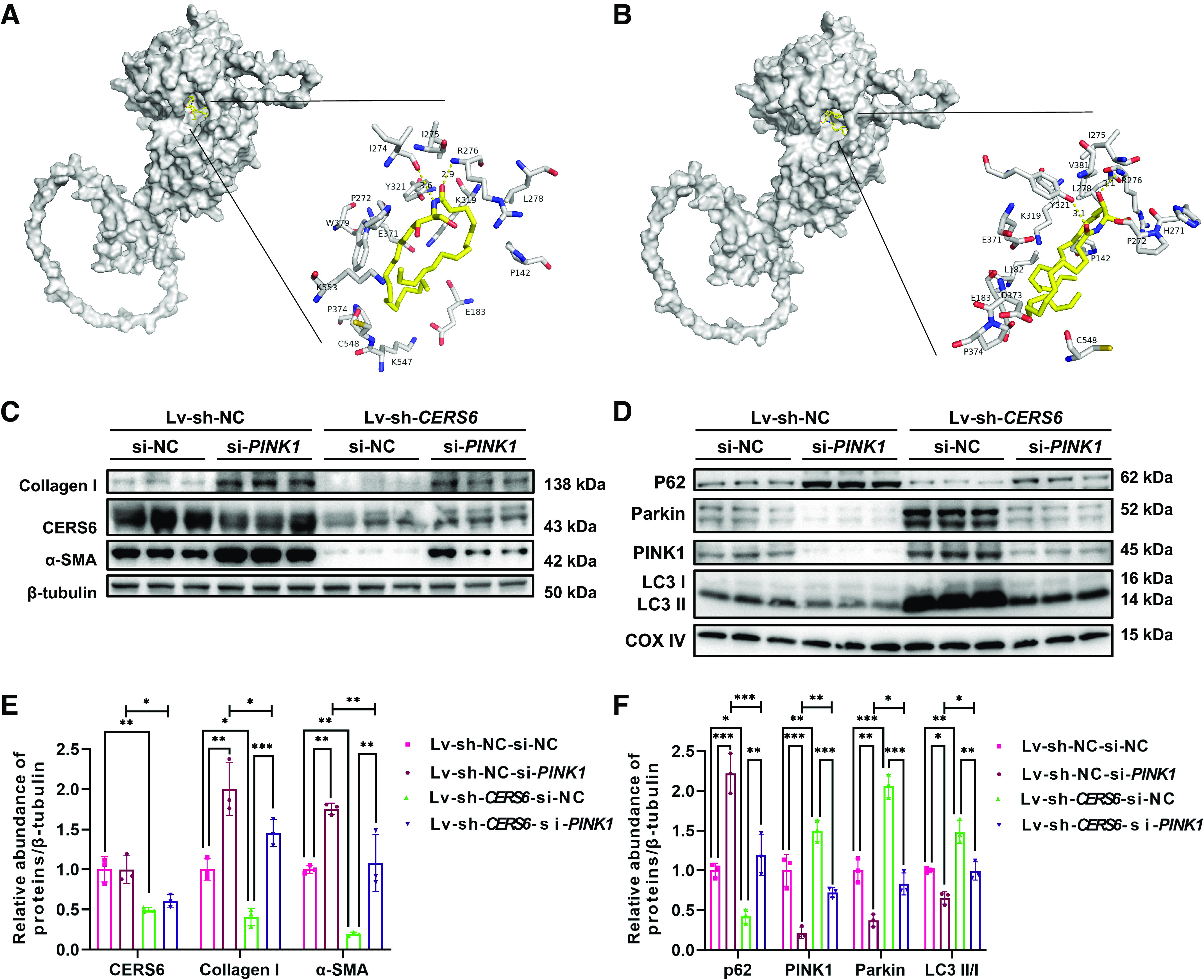
Knockdown of *CERS6* and *PINK1* in HK-2 cells could not improve mitophagy or tubulointerstitial fibrosis. Docking and virtual screening of PINK1 with CER (d18:1/14:0) (*A*) or CER (d18:1/16:0) (*B*). *C*, *E*: Western blots and quantification of Collagen I, α-SMA, and CERS6 in lv-sh-NC or lv-sh-*CERS6* HK-2 cells transfected with empty vector (si-NC) or *PINK1* siRNA (si-PINK1) plasmid under BSA-PA conditions. Data are shown as means ± SD, *n* = 3, **P < 0.01, ***P < 0.001. *D*, *F*: Western blots and quantification of p62, PINK1, Parkin, and LC3 in mitochondria isolated from HK-2 cells under each condition. Data are shown as means ± SD, *n* = 3, *P < 0.05, **P < 0.01, ***P < 0.001. BSA, bull serum albumin; CERS6, ceramide synthase 6; PINK1, PTEN-induced kinase 1; PA, paraformaldehyde.

Based on the above results, we hypothesized that mitophagy in renal tubular epithelial cells might be mediated by CERS6-derived CER and PINK1. To predict the possibility of the combination between CERS6-derived CER and PINK1 protein, we docked CER (d18:1/14:0) (Fig. 5*A*) and CER (d18:1/16:0) (Fig. 5*B*) ceramide to the PINK1 domain using Autodock Vina. The predicted results were that the binding energies of CER (d18:1/14:0) and CER (d18:1/16:0) to PINK1 were −5.4 kcal/mol and −5.3 kcal/mol, respectively, indicating that both CER had certain binding effects with PINK1. To verify whether CERS6 monitors mitophagy through a PINK1-reliant pathway, *PINK1* expression was blocked by siRNA in PA-induced CERS6-deficient HK-2 cells. Western blot analysis declared that the anti-fibrotic and mitophagy promotion effects caused by *CERS6* deficiency were repealed by pretreatment with *PINK1* siRNA, whereas the expression of CERS6 was not affected (Fig. 5, *C*–*F*). Altogether, elevated CERS6-derived CER in PA-stimulated HK-2 cells may bind to PINK1 protein, block PINK1/Parkin-mediated mitophagy, and ultimately lead to fibrosis.

## DISCUSSION

In this study, we used cell and animal models to elucidate the mechanism that knockdown of *CERS6* inhibited CER (d18:1/14:0) and CER (d18:1/16:0), which restored PINK1-mediated mitophagy, thereby improving renal interstitial fibrosis in DKD. As a product of lipid and protein metabolism, CER has been shown to accumulate in different tissues in obese or diabetic people and their animal models (23, 24). It has been shown that plasma CER (d18:1/16:0) levels are elevated in patients with DKD (14). Raichur et al. (25) screened antisense oligonucleotides that could effectively knockdown the expression of *Cers6* in vivo, and successfully knockdown the expression of *Cers6* and CER (d18:1/16:0) in obese and diabetic mice, importantly, ameliorated their insulin resistance, hyperglycemia, and obesity phenotypes. This suggests that ceramide synthesis and the CER they produce could be therapeutic targets. However, the causal relationship between this effect and the development of DKD has not been elucidated. We combined targeted mass spectrometry with molecular biological experiments and found that DKD mice’s renal cortex had increased levels of *Cers6* expression and CER(d18:1/14:0) and CER (d18:1/16:0), respectively. More importantly, the knockdown of *Cers6* in db/db mice not only revised renal interstitial fibrosis but also reduced the number of damaged mitochondria in the renal cortex and restored mitophagy. Furthermore, we knocked down *PINK1* in HK-2 cells with stable *CERS6* deficiency and found that PINK1 deficiency counterbalanced the protective effect of *CERS6* knockdown in PA-induced conditions. Our results illustrated that CERS6-derived ceramide may aggravate interstitial fibrosis in diabetic kidney disease by regulating PINK1-mediated mitophagy.

RTECs are the major consumer of ATP. Its function is closely related to mitochondrial energy production. As highly dynamic organelles, mitochondria provide energy to the cell and perform quality control, whereas defective mitochondria are disposed of through mitophagy (26). The process of mitophagy, generally regarded as one of the most important mechanisms of mitochondrial quality control, consists of phagocytosis of mitochondria and their removal by cellular autophagy mechanisms (27). Although whether autophagy plays a protective or damaging role in DKD remains to be determined, most studies have found that with the progress of DKD, mitophagy was inhibited and damaged mitochondria increased, resulting in reduced ATP production and kidney injury (28). It was shown by Livingston et al. that mitophagy plays a crucial role in the guardian effect of renal ischemic preconditioning (29). In a model of unilateral ureteral occlusion-induced acute kidney injury, Li et al. (17) found that PINK1/Parkin-mediated mitophagy deficiency in RTECs led to renal interstitial fibrosis. By improving mitophagy deficiency in DKD through Nrf2/PINK1, Xiao et al. (30) found that MitoQ, a mitochondrial antioxidant, alleviated RTEC apoptosis. These findings matched our observation that mitophagy was significantly inhibited in RTECs under DKD status while improving mitophagy deficiency in RTECs could effectively reduce damaged mitochondria and delay the development of DKD.

When the membrane potential in damaged mitochondria decreases, PINK1 recognizes and aggregates to the outer mitochondrial membrane of damaged mitochondria and recruits Parkin through PINK1-mediated phosphorylation of mitochondrial fusion protein 2, ubiquitin, and Parkin. Autophagy adaptors recognize the ubiquitin chain on mitochondrial outer membrane proteins, which is coupled by activated Parkin and binds the ubiquitinated cargo to the autophagosome by LC3 (27). Vos et al. (31) showed that CER accumulated in PINK1-deficient flies and was negatively correlated with mitochondrial β-oxidation and electron transport chain function. However, inhibition of ceramide synthesis could stimulate β-oxidation and ameliorate the damage caused by PINK1 deficiency. In the current research, consistent with previous findings, inhibition of *PINK1* expression reduced the improvement of mitophagy and fibrosis-related protein deposition caused by *CERS6* deficiency.

Interestingly, results from mass spectrometry showed an increase in several CER in the renal cortex of db/db mice, while ceramide synthesis at the transcriptional level differed only in *Cers6*, and knockdown of *Cers6* decreased several other CER simultaneously. As a result of a high-fat diet-induced obesity diet, Hammerschmidt et al. observed an increase in multiple CER. They constructed *ceramide synthase 5* or *Cers6* knockout mice and found that only knockout of *Cers6* could regulate CER (d18:1/16:0) in mitochondria and mitochondria-associated membrane structures, and ultimately improved obesity-related insulin resistance, while *ceramide synthase 5* deficiency showed no improvement (32). As reported by Turpin-Nolan et al., obese mice have elevated levels of *ceramide synthase 1* and CER (d18:1/18:0) production in their skeletal muscles. CER (d18:1/18:0) accumulation was reduced when *ceramide synthase 1* was knocked down in skeletal muscle, and the systemic glucose metabolism was enhanced in obese mice (33). Ceramide synthases may regulate CER distribution by different mechanisms in various organs under pathogenic status. Although we have no direct evidence for the interaction between CERS6-derived CER and PINK1, we found the possibility of CERS6-derived CER binding to PINK1 protein by computational docking. Meanwhile, the experimental results showed that further knockdown of *PINK1* in the *CERS6*-deficient HK-2 cells would negate the protective effect of knockdown of *CERS6*. An investigation of the molecular basis of the interaction is required in the future.

Although our study provides important insights into the role of CERS6 and ceramides in DKD, it is important to acknowledge a limitation in our research. Specifically, we did not confirm our findings in human kidney organoids, which would have provided greater translational relevance to our results. However, the lack of access to human kidney tissue specimens and the challenges associated with establishing reliable human kidney organoid models constrained our ability to conduct these experiments. Despite this limitation, our study using mouse models and in vitro cell culture systems provides important insights into the mechanisms underlying DKD and the role of ceramide synthesis in renal interstitial fibrosis. Our findings highlight the potential therapeutic relevance of targeting CERS6 and modulating ceramide metabolism in DKD. Future studies should aim to confirm and extend our findings in human kidney organoids to bridge the gap between preclinical research and clinical application. Due to resource constraints and experimental limitations, we were unable to examine mitochondrial damage in renal tubular epithelial cells upon *CERS6* knockdown in our study. Future research efforts should aim to address this limitation by conducting cell-based experiments using appropriate methodologies to directly assess mitochondrial damage in RTECs. By doing so, we can gain a more complete understanding of the relationship between CERS6, ceramides, and mitochondrial dysfunction in DKD, thereby advancing the field and potentially identifying novel therapeutic targets.

Collectively, the results of our study suggested that CERS6-derived CER (d18:1/14:0) and CER (d18:1/16:0) may inhibit PINK1/Parkin-regulated mitophagy, impeding the clearance of damaged mitochondria in RTECs, aggravating renal interstitial fibrosis and DKD. This mechanism lays the groundwork for future therapeutic strategies for patients with DKD, especially in patients with lipid metabolism abnormalities.

## DATA AVAILABILITY

The data set used and/or analyzed during the current study is available from the corresponding author upon reasonable request.

## GRANTS

This research was funded by the National Natural Science Foundation of China (82070848, to Y.X.); the Key-Area Research and Development Program of Guangdong Province (2019B020230001, to Y.X.); the Basic and Applied Basic Research Foundation of Guangdong Province (2021A1515010998, to Y.X.); Natural Science Foundation of Guangdong (2021A1515111025, to L.L.); the Basic and Application Base Research Project of Guangzhou Basic Research Plan (202201011436, to X.W.); and the President Foundation of Nanfang Hospital, the Southern Medical University (2021C029, to X.W.; 2020C010, to M.S.).

## DISCLOSURES

No conflicts of interest, financial or otherwise, are declared by the author.

## AUTHOR CONTRIBUTIONS

X.W., M.S., Y.Y., C.Z., and Y.X. conceived and designed research; X.W., M.S., X.L., C.S., S.R., W.D., J.C., T.W. performed experiments; X.W. and C.L. analyzed data; X.W., M.S., X.L., C.S., K.W., Z.Z., Y.J., J.C., and L.L. interpreted results of experiments; X.W. and M.S. prepared figures; X.W. and M.S. drafted manuscript; K.W., M.G., and C.Z. edited and revised manuscript; C.Z. and Y.X. approved final version of manuscript.
